# Gaps and Opportunities of Artificial Intelligence Applications for Pediatric Oncology in European Research: A Systematic Review of Reviews and a Bibliometric Analysis

**DOI:** 10.3389/fonc.2022.905770

**Published:** 2022-05-31

**Authors:** Alberto Eugenio Tozzi, Francesco Fabozzi, Megan Eckley, Ileana Croci, Vito Andrea Dell’Anna, Erica Colantonio, Angela Mastronuzzi

**Affiliations:** ^1^ Multifactorial and Complex Diseases Research Area, Bambino Gesù Children’s Hospital, Istituto di Ricerca e Cura a Carattere Scientifico (IRCCS), Rome, Italy; ^2^ Department of Onco Hematology and Cell and Gene Therapy, Bambino Gesù Pediatric Hospital, Istituto di Ricerca e Cura a Carattere Scientifico (IRCCS), Rome, Italy; ^3^ Department of Pediatrics, University of Rome Tor Vergata, Rome, Italy

**Keywords:** artificial intelligence, pediatric oncology, childhood cancer, machine learning, deep learning, CNS tumors

## Abstract

The application of artificial intelligence (AI) systems is emerging in many fields in recent years, due to the increased computing power available at lower cost. Although its applications in various branches of medicine, such as pediatric oncology, are many and promising, its use is still in an embryonic stage. The aim of this paper is to provide an overview of the state of the art regarding the AI application in pediatric oncology, through a systematic review of systematic reviews, and to analyze current trends in Europe, through a bibliometric analysis of publications written by European authors. Among 330 records found, 25 were included in the systematic review. All papers have been published since 2017, demonstrating only recent attention to this field. The total number of studies included in the selected reviews was 674, with a third including an author with a European affiliation. In bibliometric analysis, 304 out of the 978 records found were included. Similarly, the number of publications began to dramatically increase from 2017. Most explored AI applications regard the use of diagnostic images, particularly radiomics, as well as the group of neoplasms most involved are the central nervous system tumors. No evidence was found regarding the use of AI for process mining, clinical pathway modeling, or computer interpreted guidelines to improve the healthcare process. No robust evidence is yet available in any of the domains investigated by systematic reviews. However, the scientific production in Europe is significant and consistent with the topics covered in systematic reviews at the global level. The use of AI in pediatric oncology is developing rapidly with promising results, but numerous gaps and challenges persist to validate its utilization in clinical practice. An important limitation is the need for large datasets for training algorithms, calling for international collaborative studies.

## Introduction

Childhood cancer is one of the priorities of the World Health Organization (WHO) that in 2018 launched the WHO Global Initiative on Childhood Cancer, aiming at achieving 60% survival rate by 2030 (1). Cancer remains the leading cause of disease-related mortality among children 1 to 14 years of age (2), and in Europe, in 2020, over 15,500 children and adolescents were diagnosed with cancer, and more than 2,000 young patients died (2). Nonetheless, the fight against pediatric cancer is one of the most successful stories in medicine over the last decades, with an overall survival (OS) now exceeding 80% in high-income countries (3).

Although the concept of artificial intelligence (AI) was born decades ago (4), the increased availability of computational power at affordable cost has been a significant impulse in its application to several domains and AI may represent an efficient solution for many unmet needs in pediatric oncology. Yet, AI in medicine is underdeveloped, although the perspectives of its application are wide and very promising (5, 6). The several applications of AI in cancer are intuitive, as they may exploit all data generated by patients including the integration of next generation sequencing, the analysis of imaging and pathology, and may accelerate drug discovery (7). These assumptions represent the foundation of precision oncology, which aims at precisely targeting and characterizing individual tumor cells. However, many of these potential objectives have not yet been achieved due to a number of challenges.

Another promising application of AI regards the improvement of healthcare through process mining which may inform clinical management, and ultimately affect the quality of care (8). The large amount of data generated during the patient journey and the high variability of patterns of care represent an ideal area for creating AI models that can support process and resource optimization. This is particularly true in pediatric oncology where complexity of care is high due to severity of cancer and comorbidity.

Although technical solutions for the development of AI applications are largely available, AI algorithms require large amounts of data from interoperable datasets for a widespread application of AI that achieves a high accuracy (9). Moreover, regulations regarding AI systems require appropriate risk management and testing, technical robustness with sufficient data training, and clear plans on data governance, transparency, human control and cyber security, which may be hard to obtain without a multidisciplinary approach and an investment of resources (10).

In 2021, the European Union (EU) Commission issued the Europe’s Beating Cancer Plan, including the flagship Helping Children with Cancer Initiative, which highlights the value of real word data and artificial intelligence as potential tools for cancer prevention and care (11). The EU Commission also underlined in its Review of the Coordinated Plan on AI, the value of artificial intelligence in supporting cancer diagnosis and therapy through the creation of appropriate infrastructures and digital solutions (ANNEXES to the Communication from the Commission to the European Parliament, the European Council, the Council, the European Economic and Social Committee and the Committee of the Regions Fostering a European approach to Artificial Intelligence) (12).

In this scenario of potential rapid development, designing plans and addressing priorities in AI applications for pediatric oncology requires an analysis of the current activities to identify the achievements and the gaps in this field, including clinical and management issues. To this aim, we drew the state of the art in the field of AI applied in pediatric oncology through a systematic review of reviews. In addition, to describe the existing scientific trends in Europe, we performed a bibliometric analysis of publications authored by European authors in the same field.

## Methods

We performed a systematic review of systematic reviews in the field of AI applied on pediatric oncology. To this end, we set up a search query based on a published strategy of the Cochrane Childhood Cancer for PubMed (13) and a query translating technical terms relevant to AI and radiomics. This query was adapted and submitted to Web of Science and PubMed limiting the search to reviews and to the time from January 2000 to September 2021. The detailed search query is described in Appendix 1 (available as Supplementary materials). We manually selected those reviews which included studies a) with individuals below 18 years of age; b) which focused on tumors typical of pediatric age; c) reporting quantitative results. We excluded the publications on tumors of adulthood even if including some patients < 18. The list of included tumors is available in the Supplemental material. Where the age range of the studies was not indicated, papers containing information potentially applicable in pediatrics were included, although some studies were presumably conducted in adult populations (eg, CNS tumors). From the publications selected, we manually extracted the following information: disease on which AI was applied, the number of studies included in the review, the type of AI intervention, its aim, and the data source used for AI development. We also summarized the key findings of each review. Finally, we manually extracted the studies included in these reviews and submitted them to Scopus to calculate the proportion of those including authors with a European affiliation. As in this review we covered a broad range of topics in AI and pediatric oncology, we could not apply the recommendations for reporting systematic reviews according to the Preferred Reporting Items for Systematic Reviews and Meta-Analyses (PRISMA) (14).

In addition, we performed a bibliometric analysis on available publications on AI and pediatric oncology from a search query submitted to the Web of Science platform. We used the same search string adopted for searching the reviews, but we selected the original publications instead. We then manually reviewed the selected records and included the original publications that included quantitative results on AI interventions applied to pediatric cancer. From these publications, we manually extracted information on the disease in which AI was applied, the aim of the work, the data source used for the AI application, and if the performance of the AI algorithm was compared with a natural human. Finally, we extracted information on the funding source in Scopus.

The bibliometric analysis of this dataset included the annual volume of scientific publications, the collaboration network based on Authors’ affiliation, and the volume of publications by journal. For this purpose, we used the Biblioshiny platform (15). Descriptive statistics were performed through STATA 17.0 (Stata Corporation, College Station, Texas).

## Results

### Systematic Review of Systematic Reviews

We found 330 unique records from the combination of results from Web of Science and PubMed. We manually selected those reviews which included studies a) with individuals below 18 years of age; b) which focused on tumors typical of pediatric age; c) reporting quantitative results. We excluded the publications on tumors of adulthood even if including some patients < 18. The list of excluded tumors is available in the Supplemental material.

**Figure 1:**
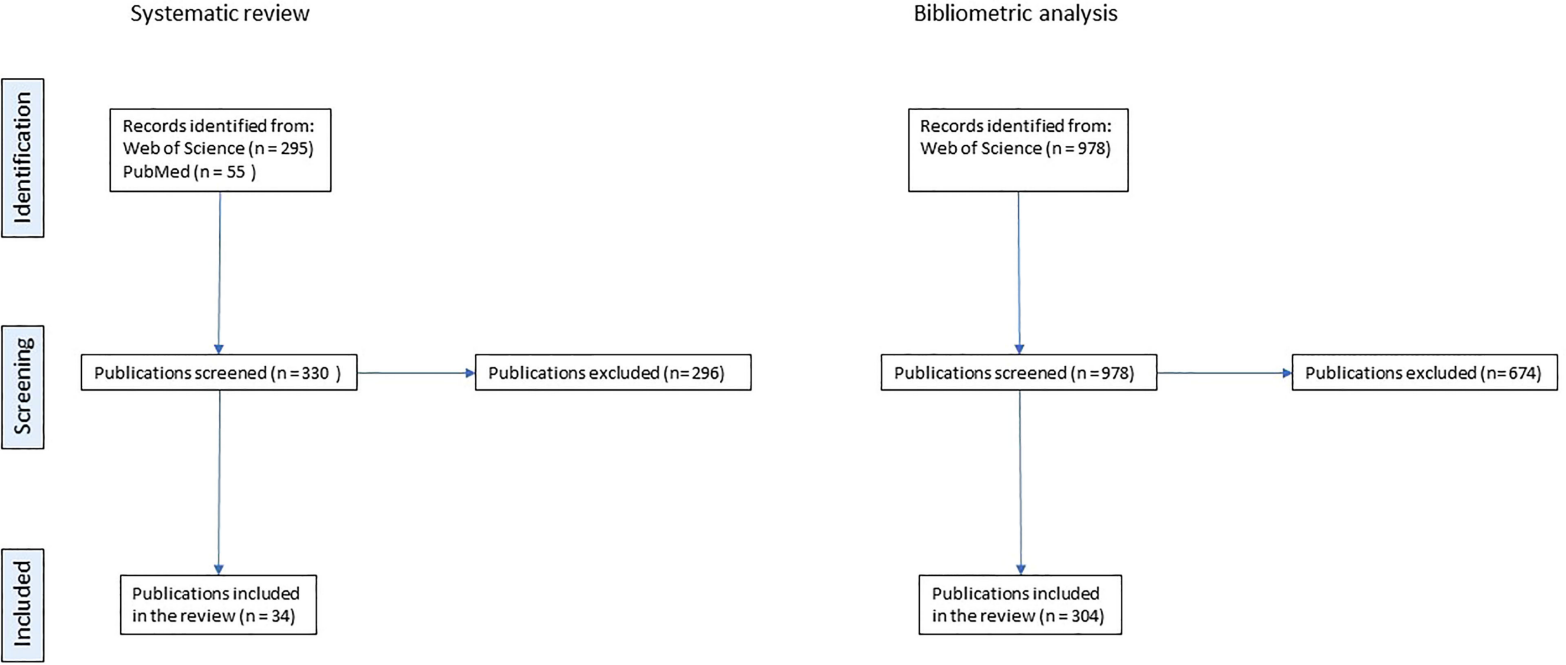
PRISMA flowchart describing the selection process for systematic reviews and for original papers included in the bibliometric analysis.

Finally, 25 records were eligible to be included in the review (**Figure 1**).

**Table 1** shows a summary of the 25 included reviews by disease and type of data used for AI application (16–40).

**Table 1:** Summary of reviews included in the systematic review.

Reference	Year	Number Of Iincluded Studies	Cancer Group	Data Source	AI Application	AIm	Key Findings
Katsila T, et al. (16)	2017	220	CNS tumors	MRI	Radiomics, pharmacometabolomics	Diagnosis	Radiomics: (i) tumor grading needs to be better-refined, (ii) diagnostic precision should be improved, (iii) standardization in radiomics is lacking, and (iv) quantitative radiomics needs to prove clinical implementation. Pharmacometabolomics: data regarding this topic in CNS tumors are scarce.
Nguyen A V, et al. (17)	2018	8	CNS tumors	MRI	Machine learning algorithms	Differential diagnosis	In differentiation of primary central nervous system lymphoma from glioblastoma on imaging, ML performed well with the lowest reported AUC being 0.878. In studies in which ML was directly compared with radiologists, ML performed better than or as well as the radiologists. However, when ML was applied to an external data set, it performed more poorly.
Sarkiss CA, et al. (18)	2019	29	CNS tumors	Diagnostic images, gene expression	Machine learning algorithms	Diagnosis, prognosis	ML can predict patient outcomes, with a sensitivity range of 78%–98% and specificity range of 76%–95%. ML based algorithms show accuracy in diagnosing low-grade versus high-grade gliomas, ranging from 80% to 93% and 90% for diagnosing high-grade glioma versus lymphoma.
Sohn CK, et al. (19)	2020	5	CNS tumors	MRI	Radiomics	Diagnosis	The pooled sensitivity when diagnosing HGG was higher (96% (95% CI: 0.93, 0.98)) than the specificity when diagnosing LGG (90% (95% CI 0.85, 0.93)). Heterogeneity was observed in both sensitivity and specificity. Metaregression confirmed the heterogeneity in sample sizes (p = 0.05), imaging sequence types (p = 0.02), and data sources (p = 0.01), but not for the inclusion of the testing set (p = 0.19), feature extraction number (p = 0.36), and selection of feature number (p = 0.18). The results of subgroup analysis indicate that sample sizes of more than 100 and feature selection numbers less than the total sample size positively affected the diagnostic performance in differentiating HGG from LGG.
Park JE, et al. (20)	2020	51	CNS tumors	MRI	Radiomics	Quality of radiomics studies	Prognostic/predictive studies received higher score than diagnostic studies in comparison to gold standard (P <.001), use of calibration (P = .02), and cut-off analysis (P = .001). The quality of reporting of radiomics studies in neuro-oncology is currently insufficient.
Bhandari AP, et al. (21)	2020	9	CNS tumors	MRI	Convolutional Neural Networks algorithms	Brain tumors segmentation	Only one study used a training set from their own institution. Specifics of convolution layers (i.e. filtration of images) were not detailed extensively. The majority of overfitting was done *via* down sampling. CNN has a role in segmentation of brain tumors such as glioblastoma and lower grade astrocytomas.
Bhandari AP, et al. (22)	2020	14	CNS tumors	MRI	Radiomics	Classification	The best classifier of IDH status was with conventional radiomics in combination with convolutional neural network–derived features (AUC = 0.95, 94.4% sensitivity, 86.7% specificity). Optimal classification of 1p19q status occurred with texture-based radiomics (AUC = 0.96, 90% sensitivity, 89% specificity). A meta-analysis showed high heterogeneity due to the uniqueness of radiomic pipelines.
Booth TC, et al. (23)	2020	20	CNS tumors	MRI, PET	Radiomics	Diagnosis, prognosis and treatment response	Much research is applied to determining molecular profiles, histological tumor grade, and prognosis using MRI images acquired at the time that patients first present with a brain tumor. Although pioneering, most of the evidence is of a low level, having been obtained retrospectively and in single centers.
Tewarie IA, et al. (24)	2021	27	CNS tumors	Genomics, MRI, clinical information, histopathology, pharmacokinetics	Algorithmic prognostic models	Prognosis	The included studies developed and evaluated 59 models, of which only seven were externally validated in a different patient cohort. The predictive performance among these studies varied widely according to the AUC (0.58–0.98), accuracy (0.69–0.98), and C-index (0.66–0.70). However, none of these models has been implemented into clinical care
van Kempen EJ, et al. (25)	2021	17	CNS tumors	MRI	Machine learning algorithms	Prediction of glioma genotype	Meta-analysis showed excellent accuracy for all subgroups, with the classification of 1p/19q codeletion status scoring significantly poorer than other subgroups (AUC: 0.748, p = 0.132). Classification of IDH mutation shows an overall AUC of 0.909 (95%-CI: 0.867–0.951). AUC of MGMT promoter methylation status was estimated as 0.866 (95%-CI: 0.812–0.921). There was considerable heterogeneity among some of the included studies.
van Kempen EJ, et al. (26)	2021	8	CNS tumors	MRI	Machine learning algorithms -based glioma segmentation tools	Accuracy of brain tumor segmentation	Overall, the MLAs from the included studies showed an overall dice similarity coefficient (DSC) score of 0.84 (95% CI: 0.82–0.86). In addition, a DSC score of 0.83 (95% CI: 0.80–0.87) and 0.82 (95% CI: 0.78–0.87) was observed for the automated glioma segmentation of the high-grade and low-grade gliomas, respectively. However, heterogeneity was considerably high between included studies, and publication bias was observed
Al-Galal SAY, et al. (27)	2021	92	CNS tumors	MRI	Deep learning techniques for classification and segmentation	Brain tumors segmentation, classification	The significant advantage of the techniques of DL is its computability and consistency with many conventional techniques. DL methods focusing on convolutional neural networks (CNN) are more applicable to all sub-fields of medical image processing, such as classification, identification, and segmentation.
Buchlak QD, et al. (28)	2021	153	CNS tumors	MRI	Machine learning for diagnosis and classification	Diagnosis, classification	Model performance of machine learning was generally strong (AUC = 0.87 ± 0.09; sensitivity = 0.87 ± 0.10; specificity = 0.0.86 ± 0.10; precision = 0.88 ± 0.11). Convolutional neural network, support vector machine and random forest algorithms were top performers.
Jian A, et al. (29)	2021	44	CNS tumors	MRI	Radiomics	Diagnosis, prediction of molecular markers	The pooled sensitivity and specificity for predicting isocitrate dehydrogenase (IDH) mutation in training datasets were 0.88 (95% CI 0.83-0.91) and 0.86 (95% CI 0.79-0.91), respectively, and 0.83 to 0.85 in validation sets. Use of data augmentation and MRI sequence type were weakly associated with heterogeneity. Both O6-methylguanine-DNA methyltransferase (MGMT) gene promoter methylation and 1p/19q codeletion could be predicted with a pooled sensitivity and specificity between 0.76 and 0.83 in training datasets.
Tabatabaei M, et al. (30)	2021	18	CNS tumors	MRI	Radiomics	Classification	Results appear promising for grade prediction from MR images using the radiomics techniques. However, there is no agreement about the radiomics pipeline, and the prior studies are very heterogeneous regarding the software used, the number of extracted features, MR sequences, and machine learning technique. Before the clinical implementation of glioma grading by radiomics, more standardized research is needed.
d’Este SH, et al. (31)	2021	14	CNS tumors	PET	Combination of multimodality imaging with AI	Defining tumor infiltration by imaging	All studies concluded their findings to be of significant value for future clinical practice. Diagnostic test accuracy reached an area under the curve of 0.74–0.91 reported in six studies. When AUC is not provided, a sensitivity of 80.0%–100% and a specificity of 69.2%–100%, an accuracy of 78%–81.8% and a Pearson’s correlation coefficient of 0.74–0.88 were found.
Zhong J, et al. (32)	2020	12	Bone and soft tissue sarcomas	MRI, PET	Radiomics	Quality of radiomics studies; prognosis	Median Radiomics Quality Score: 5 (–5, 16). None of the included studies performed a phantom study. Most included studies were regarded as having a moderate risk of bias. Meta-analysis of radiomics studies predicting osteosarcoma response to neoadjuvant chemotherapy showed a high diagnostic odds ratio 43.68 (95% CI 13.50–141.31) and the area under the curve was 0.91 (95% CI 0.89–0.94), which indicates a high diagnostic performance. The overall scientific quality of radiomics studies in osteosarcoma was insufficient and heterogeneity of studies was high suggesting that radiomics is far from a clinical applicable tool.
Crombé A, et al. (33)	2020	52	Bone and soft tissue sarcomas	MRI, PET, CT, Ultrasound	Radiomics	Quality of radiomics studies	None of the study did a test-retest analysis of the radiomic features nor a phantom study. Only two studies were prospectively designed. Thirty-eight out of 52 (73.1%) studies did not validate their results on an independent cohort. Median Radiomics Quality Score: 4,5 (–7, 17). The median number of extracted radiomic features was 65 (range: 9–210105). Twelve of the 52 (23.1%) studies only performed an exploratory univariate analysis. Overall, our results show that the quality of sarcoma radiomics studies is low on average, which may hamper the reproducibility of radiomics models on external cohorts and, therefore, practical applications of these models.
Gitto S, et al (34)	2021	49	Bone and soft tissue sarcomas	MRI, CT	Radiomics	Reproducibility and prediction of diagnosis	Eighteen (37%) of the 49 studies included a reproducibility analysis of the radiomic features in their workflow. The intraclass correlation coefficient (ICC) was the statistical method used in most of the papers reporting a reproducibility analysis. At least one machine learning validation technique was used in 25 (51%) of the 49 papers. A clinical validation of the radiomics-based prediction model was reported in 19 (39%) of the 49 papers. The quality of sarcoma radiomics studies is low, which may hamper performance generalizability of radiomic models on independent cohorts and, consequently, their practical application.
Wang H, et al. (35)	2020	45	Lymphomas	MRI, CT, PET	Radiomics	Diagnosis, prognosis, quality of radiomics studies	Radiomics features can be used to effectively differentiate lymphoma from another disease (AUC values of the studies ranged from 0.730 to 1.000). Radiomics features are prognostic predictors for the outcome of patients with several types of lymphoma. However, the quality of published radiomics studies in lymphoma has been suboptimal to date.
Frood R, et al. (36)	2021	41	Lymphomas	PET; CT	Quantitative imaging parameters derived from pretreatment FDG PET/CT; radiomics	Prognosis, treatment outcome	Significant predictive ability was reported in 5/20 DLBCL studies assessing SUVmax (PFS: HR 0.13–7.35, OS: HR 0.83–11.23), 17/19 assessing metabolic tumor volume (MTV) (PFS: HR 2.09– 11.20, OS: HR 2.40–10.32) and 10/13 assessing total lesion glycolysis (TLG) (PFS: HR 1.078–11.21, OS: HR 2.40–4.82). Significant predictive ability was reported in 1/4 HL studies assessing SUVmax (HR not reported), 6/8 assessing MTV (PFS: HR 1.2–10.71, OS : HR 1.00–13.20) and 2/3 assessing TLG (HR not reported). There are 7/41 studies assessing the use of radiomics (4 DLBCL, 2 HL); 5/41 studies had internal validation and 2/41 included external validation. All studies had overall moderate or high risk of bias.
Badrigilan S, et al. (37)	2021	30	Head and neck cancer	MRI	AI assisted classification and segmentation	Tumor segmentation, classification	The overall performance of DL models for the complete tumor in terms of the pooled Dice score, sensitivity, and specificity was 0.8965 (95% confidence interval (95% CI): 0.76–0.9994), 0.9132 (95% CI: 0.71–0.994) and 0.9164 (95% CI: 0.78–1.00), respectively. The DL methods achieved the highest performance for classifying three types of gliomas, meningioma, and pituitary tumors with overall accuracies of 96.01%, 99.73%, and 96.58%, respectively. Stratification of glioma tumors by high and low grading revealed overall accuracies of 94.32% and 94.23% for the DL methods, respectively.
Carbonara R, et al. (38)	2021	8	Head and neck cancers	MRI, PET, CT	Radiomics	Prediction of radiation-induced side effects	Published radiomic studies provide encouraging but still limited and preliminary data that require further validation to improve the decision-making processes in preventing and managing radiation-induced toxicities.
Gupta V, et al. (39)	2020	27	HSCT	Clinical data, imaging, genomic and demographic data	Machine learning techniques	Prognosis	The majority of studies used supervised ML, related to post-HSCT complications, but were limited by small numbers of patients. None of the studies provided robust evidence to determine an optimal ML technique for HSCT or minimal number of variables required to build predictive models. However, our results suggest that ADT could be applicable in HSCT settings due to their interpretability.
Salah HT, et al. (40)	2019	23	Leukemia	Microscopy, flow cytometry	Machine learning techniques	Diagnosis	Multiple studies have applied ML tools on leukemia diagnosis. Some studies have reached high classification accuracy. Nevertheless, literature presented in this review illustrates the need for multiple future directions.

AUC, Area Under the Curve; DL, Deep Learning; DLBCL, Diffuse Large B Cell Lymphoma; HL, Hodgkin Lymphoma; HR, Hazard Ratio; CI, Confidence Interval; HGG, High Grade Glioma; LGG, Low Grade Glioma, MLAs, Machine Learning Algorithms; PFS, Progression free survival.

Although we covered a 20-year time in the search strategy for systematic reviews, none of them was published before 2017, indicating only a recent proliferation of original publications in this field. The included reviews included heterogeneous publications and rarely showed a meta-analysis with a quantitative summary of available evidence. The total number of studies included in the reviews was 674, among them, 224 (33.2%) included an author with a European affiliation.

Most available reviews focused on the use of diagnostic images, particularly radiomics. Only 4/25 reviewed the use of data sources other than diagnostic images (namely pharmacokinetics, histopathologic, genomic, and demographic data) for the development of AI applications. While, the use of AI systems to optimize highly repetitive processes (such as image segmentation) was widely investigated, an emerging trend involved the use of radiomic features in predicting the histological and molecular classification of tumors, with the aim to reduce the invasiveness of the diagnostic process.

Sixteen out of the 25 reviews focused on central nervous system (CNS) tumors, and seven of them investigated the performance of radiomics. The majority of these reviews were about the performance of AI applications in diagnosis, segmentation and classification, while 4 investigated their prognostic value. Finally, one review focused on the definition of tumor infiltration, and one on quality of radiomics studies. All reviews on CNS tumors concluded for a good performance of AI applications for the specific aim of the review. In particular, in the review by *van Kempen et al.*, *Jian et al.*, and *Bhandari et al.*, the ability to predict the genomic profile of glioblastoma, namely IDH status, MGMT promoter methylation status, and 1p/19q codeletion status, was investigated with promising perspectives (22, 25, 29). A satisfactory accuracy was also found in predicting the prognosis of patients, with a sensitivity range of 78%–98% and specificity range of 76%–95% reported in the review by *Sarkiss et colleagues* (18). Of note, the genomic features investigated by these studies are frequent in the adult population but rare in childhood, since they were found virtually in adolescents only (41, 42).

Three out of the 25 reviews were on AI applications for bone and soft tissues sarcomas and focused on quality and reproducibility of radiomics. These reviews agreed in concluding that the quality of radiomics studies is still low and that this may hamper their reproducibility and practical clinical application.

Two reviews were on lymphomas and focused on radiomics for diagnosis or prediction of outcome. Although the performance of radiomics was good, the quality of the studies was suboptimal and the risk of bias in these studies was moderate or high.

Two reviews on head and neck cancers evaluated the performance of radiomics and other AI applications: one on tumor segmentation and classification of tumors showed a high performance of AI applications, the other investigated the prediction of radiation-induced side effects through radiomics and showed the existence of preliminary data only that need validation. Again, although head and neck cancers are rare in childhood, these studies were included because one includes brain tumors, which are relatively frequent in children, while the other focuses on radiation damage, an important issue especially during the developmental stage.

The remaining reviews focused on AI applications for predicting prognosis in hematopoietic stem cell transplantation (HSCT) that yielded only weak evidence supporting machine learning (ML) techniques mainly due to small sample size, and AI applications for diagnosis of leukemia (mostly qualitative) that recommended further validation of the models. None of the reviews focused on AI applications for process analysis of patients with cancer.

Almost all the reviews underlined the limitations of the investigated studies due to small data samples for training, heterogeneous methodologies, lack of external validation, and questionable quality of the available papers included in the reviews.

### Bibliometric Analysis

We found 978 records, and we selected 304 for the analysis by manual revision applying the same selection criteria used for the reviews. Bibliometric data were extracted from the selected publications and processed to obtain information on temporal trends of publications, most used scientific journals for publication, and geographic trends according to authors’ affiliation. Manually annotated information allowed for the analysis of most frequent diseases, scope, data source, comparison of performance with humans, and funding source.

**Figure 2:**
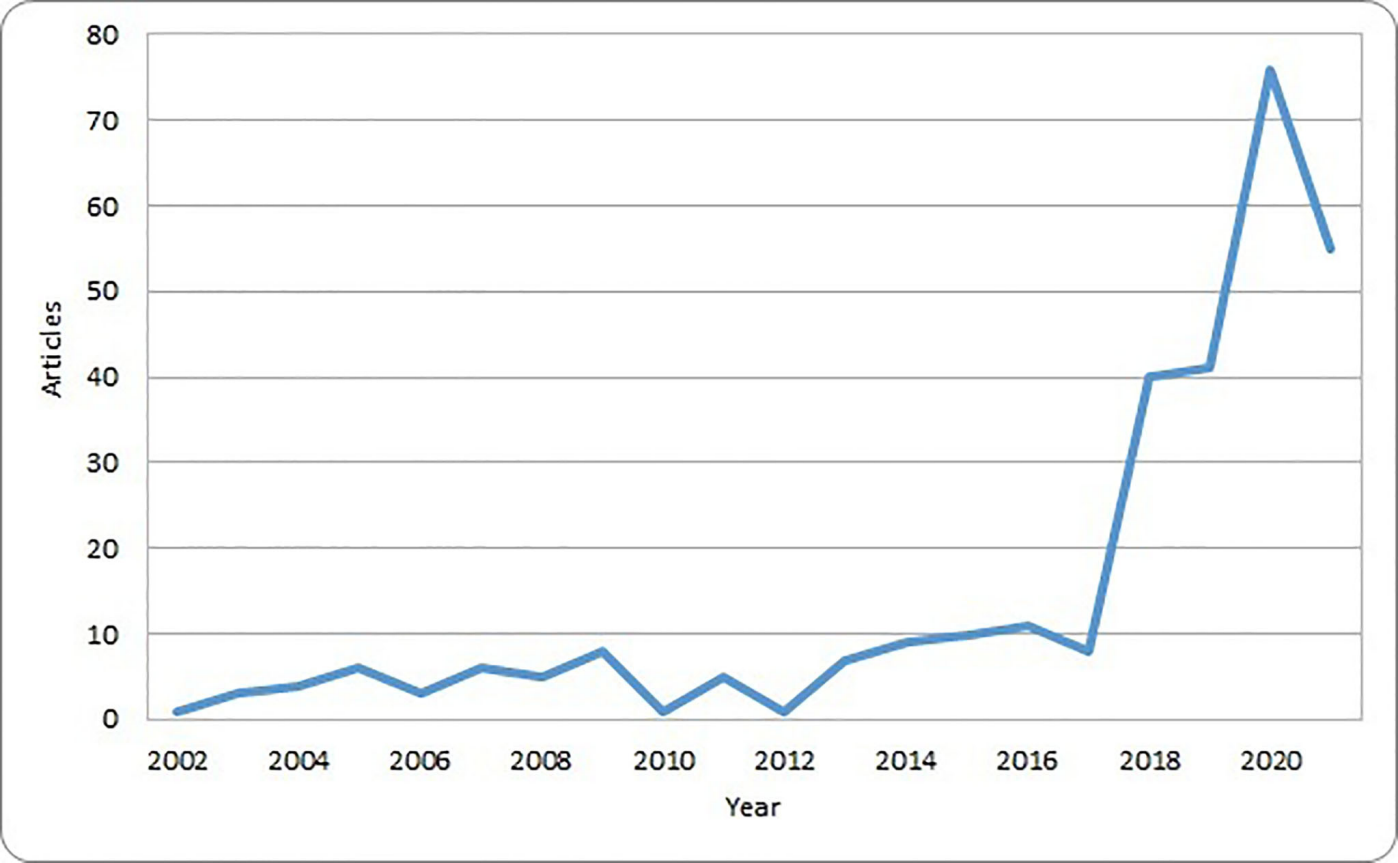
Number of original papers included in the bibliometric analysis by year of publication.

These original papers were published in 172 different journals and had an average number of citations of 32.9. The number of articles regarding AI and pediatric cancer has been modest until 2017, and then sharply raised with a nearly tenfold increase in 2020 compared with 2017 (**Figure 2**). This trend corresponds to the increasing availability of computational resources and the popularity of AI solutions in other healthcare domains and parallels the availability of systematic reviews.

Most of the scientific publications in the field of AI and pediatric cancer are available in journals that are not specific for oncology such as Scientific Reports and PLoS One (**Table 2**). Of note, most papers published before 2015 were in generic scientific journals or those about medical imaging.

**Table 2:** Scientific journals hosting original papers on AI in pediatric oncology.

Sources	n. articles	h_index	g_index	m_index	TC	NP	PY_start
PLOS ONE	10	1	1	0,250	107	1	2018
CANCERS	8	1	1	0,250	13	1	2018
IEEE ACCESS	8	2	2	0,250	63	2	2014
JOURNAL OF MEDICAL IMAGING	8	1	1	0,167	35	1	2016
IEEE TRANSACTIONS ON MEDICAL IMAGING	7	1	1	0,059	9	1	2005
MEDICAL HYPOTHESES	7	1	1	0,500	1	1	2020
SCIENTIFIC REPORTS	7	1	1	0,077	55	1	2009
APPLIED SCIENCES-BASEL	6	4	6	1,000	86	6	2018
COMPUTER METHODS AND PROGRAMS IN BIOMEDICINE	6	1	1	0,071	120	1	2008
SENSORS	6	4	4	0,222	270	4	2004
BMC BIOINFORMATICS	5	1	1	0,250	5	1	2018
COMPUTERIZED MEDICAL IMAGING AND GRAPHICS	5	1	1	1,000	3	1	2021
COMPUTERS IN BIOLOGY AND MEDICINE	5	2	2	0,125	156	2	2006
CYTOMETRY PART A	5	1	1	0,333	1	1	2019
ARTIFICIAL INTELLIGENCE IN MEDICINE	4	3	3	0,750	48	3	2018
BIOMEDICAL SIGNAL PROCESSING AND CONTROL	4	1	1	0,250	5	1	2018
DIAGNOSTICS	4	2	2	0,333	23	2	2016
NMR IN BIOMEDICINE	4	1	1	0,333	8	1	2019
AMERICAN JOURNAL OF NEURORADIOLOGY	3	1	1	0,500	1	1	2020
BIOLOGY DIRECT	3	2	3	0,182	19	3	2011

The Table includes the first 20 journals in order of number of publications. H-index: The Hirsch index (H-index) is a journal’s number of published articles (h), each of which has been cited in other papers at least h time(s). m-index: The m-index is defined as H/n, where H is the H-index and n is the number of years since the first published paper of the journal. The g-index is an improvement of H-index. TC, Total number of Citations; NP, Net Production; PY_start, starting year of the journal.

Not only the scientific production included in the analysis was scattered through several different scientific journals, but also according to the bibliometric indexes, the number of citations was modest, being higher for those journals that started to host publications on AI in pediatric oncology in early years.

**Figure 3:**
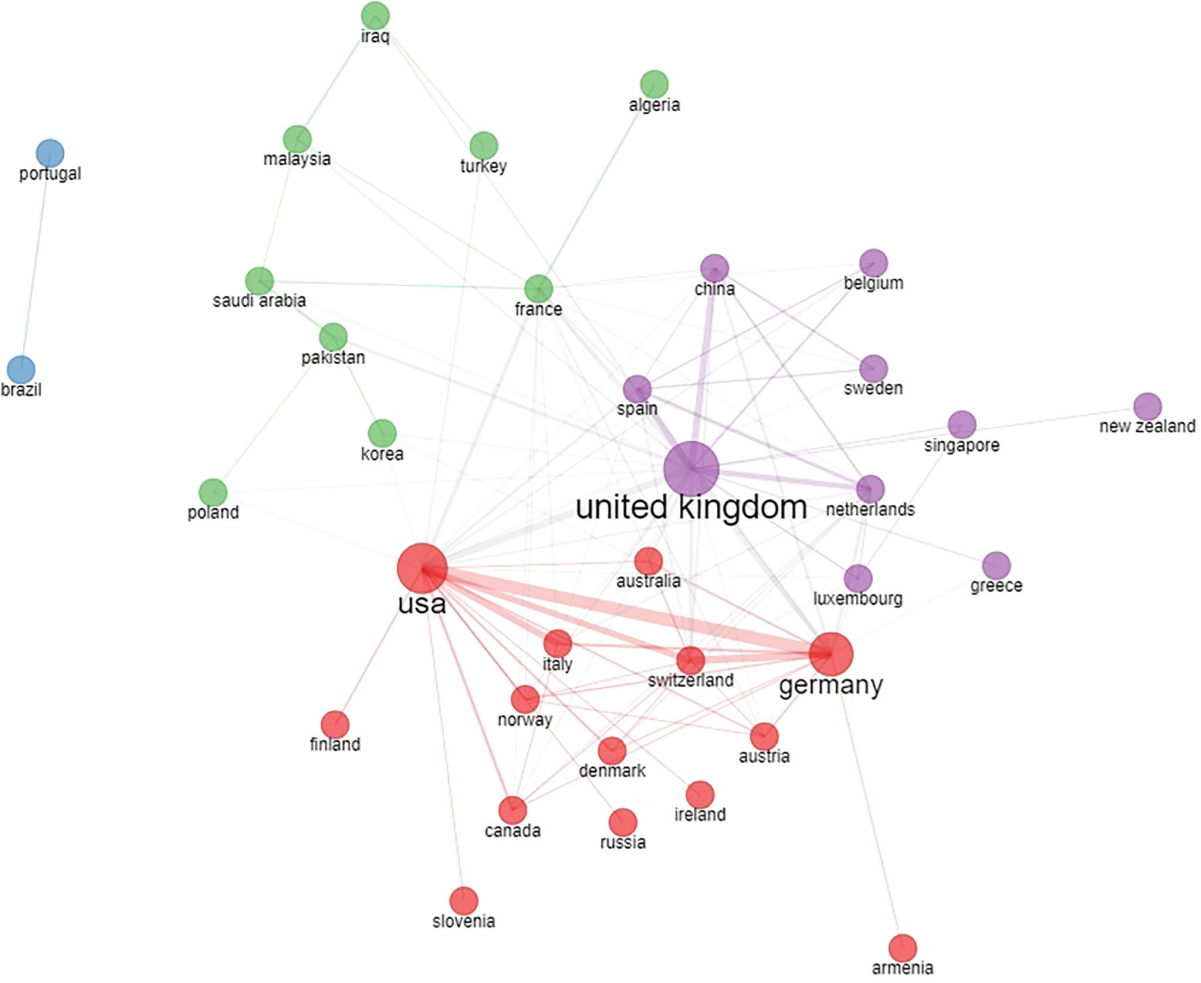
Country collaboration network based on country of authors in the original papers included in the bibliometric analysis.

The most represented author countries were the UK, Germany and Spain, accounting for 37% of all authors. International collaborations between different countries based on the affiliation of authors is shown in **Figure 3**.

Separate networks including different European countries exist with dense collaboration links. The most evident cluster is between the USA, Germany, several other European countries and Canada (**Figure 3** - red cluster). A second collaboration network is evident among the UK, China and other various countries (**Figure 3** - purple cluster). Additional collaboration networks include France and other Mediterranean and Eastern countries (**Figure 3** - green cluster), Portugal, and Brazil (**Figure 3** - blue cluster).

The distribution of clinical diseases in which the AI intervention was applied is illustrated in **Table 3**. The majority of AI applications studied in selected papers were on brain tumors and leukemia (241/334). However, the list of childhood tumors in these papers does not include many common oncologic diagnoses.

**Table 3:** Distribution of diagnosis and source of information for the AI application in publications selected for the bibliometric analysis.

	Images		Omics		Hystopathology/Blood		Other	
	n	%	n	%	n	%	n	%
CNS tumor (n=186)	146	78.5	20	10.8	17	9.1%	6	3.2
Leukemia (n=55)	1	1.8	22	40.0	27	49.1	7	12.7
Lymphoma (n=24)	6	25.0	10	41.7	6	25.0	2	8.3
Neuroblastoma (n=15)	4	26.7	10	66.7	2	13.3	0	–
Bone and soft-tissue sarcoma (n=13)	0	–	4	30.8	8	61.5	1	7.7
Wilms Tumor (n=11)	7	63.4	3	27.3	1	9.1	0	–
Hematopoietic stem cells transplantation (n=3)	0	–	1	33.3	0	–	2	66.7
Other tumors (n=27)	6	22.2	16	59.3	5	18.5	1	3.7

Each publication may include different data sources for the development of AI applications and may focus on more than one disease group.

When looking at the scope of the AI application in these papers, the domains in which AI has been studied in pediatric cancer included most frequently classification (109/304, 35.9%) and diagnosis (80/304, 26.3%) of pediatric cancer. Much less frequently, the papers focused on AI for planning treatment (20/304, 6.3%). Some papers also had multiple scopes (2.0%). We also investigated which data source was used to train the AI intervention in each of the papers under review (**Table 3**). The large majority of existing papers focused on AI applied to diagnostic images in brain cancer, followed by studies using -omics and other data sources on histopathology or blood. The most frequent category of data used in AI applications is diagnostic images, particularly magnetic resonance imaging (MRI). Some data sources, such as ultrasound images and data from metagenomics, are less frequent in these reviewed papers. Multiple data sources were rarely combined in the European reviewed papers accounting for only 5% only of the total publications. The most frequent combinations were different diagnostic images sources such as MRI, computed tomography (CT) and positron emission tomography (PET). None of the reviewed publications investigated the use of AI in process analysis of management of pediatric oncologic diseases

We also explored how frequently the AI application was compared to human capacity in terms of concordance and accuracy for external validation. The total number of papers including such a comparison with a human counterpart was 31/304 papers (10.2%). Finally, information regarding the use of any research funding was available in 294/304 papers; among them, 173 (58.8%) acknowledged a funding source for the research activity and 40 of them (13.6%) reported a European funding source, while the remaining 121 (41.2%) we not supported by any research fund. European funding supporting the work reported in these publications were from the FP6, FP7, Horizon 2020, ERDF, and ESF Programmes.

## Discussion

We searched for existing evidence and the trends in publications regarding the application of AI in pediatric tumors. Our work, which combined a systematic review of systematic reviews and a bibliometric analysis, applied a detailed search string to find the existing contributions in the domain of AI and pediatric tumors screening the existing publications over a long period of time. Although the inclusion of other publication sources than Web of Science and PubMed databases may have increased the number of publications in this field, our analysis has captured the vast majority of existing scientific production in the field of AI and pediatric oncology.

Our review focused on Europe, showed that AI in pediatric oncology is still in its infancy and, although many publications on this topic have been issued, the available evidence is still poor and mostly limited to proof-of-concept studies. No robust evidence is available yet in any of the clinical domains investigated in the available systematic reviews. On the other hand, the scientific production generated in Europe is significant and consistent with the topics covered in systematic reviews at the global level. Most of the literature generated in Europe is on AI applications for imaging in brain cancer. Radiomics, the most developed area of research, still suffers from a low degree of reproducibility and repeatability due to the paucity of existing studies and the rarity of multicentric initiatives in this field (43). However, there is a growing body of evidence exploring the use of AI in other cancers, although studies of AI applications are much more limited. Various reasons may explain this discrepancy. First, CNS tumors, although representing the most common solid neoplasms in childhood, are not exclusive to this age group since a significant percentage of diagnoses occur in adulthood. In contrast, other pediatric solid tumors are rarely found in adulthood, making it more difficult to have large enough cohorts for algorithm training. Similarly to CNS tumors, leukemias can also be found among adults, albeit with different phenotypic characteristics; therefore, one would expect more studies regarding the use of AI for these malignancies. Conversely, leukemias do not benefit from diagnostic images in which AI is mostly applied. Several authors, however, have explored the use of AI to integrate different data sources particularly for prognostic purposes. Finally, at a more general level, AI may help shorten the time consumed in repetitive tasks and this may explain why automatic tumor segmentation is a frequent topic of research.

Although the scientific production on AI and pediatric cancer significantly increased in the last years, still most of published works remain proofs of concept. Indeed, most studies used datasets from single centers, with a small sample size, and did not perform any external validation of their model, limiting their applicability and generalizability. Data-sharing privacy concerns and several other barriers preventing data linkage and multicentric collaborations result in low inter- and intra-observer variability of AI algorithms and ultimately decrease their generalizability. Moreover, ensuring external validation through comparison of AI algorithms with humans, remains essential.

Our focus on Europe showed a very active network of collaborating centers across countries in Europe and other continents. According to our bibliometric analysis, Germany and the UK, two among the most represented countries in the field investigated in this review, have strong collaborations with the US and China that co-authored several published papers. Finally, almost half of the publications from European authors included in this review did not acknowledge any funding source for their research suggesting that many of these studies are based on single center initiatives. Although authors may have missed to report a funding source, only 13% of these studies acknowledged funding from research projects of the EU Commission. The recent Horizon Europe research programme already includes several calls for proposals on AI and pediatric cancer, which hopefully will be instrumental to fill in the existing gaps in this field and to explore the existing opportunities. Several topics in this respect deserve attention.

Cancer in children may result from genetic changes that are currently unknown, linked either to inherited genetic changes or exposure to diagnostic or therapeutic radiation (44). In essence, identification of conditions that can predispose to cancer, or polymorphisms of different genes that, if associated with each other, can increase the risk of neoplasms, represent a priority in pediatric oncology. AI can help to identify high-risk populations and prescribe the most appropriate screening test for each individual (45).

In terms of diagnostic strategies, minimally invasive diagnostic tools with a broadened spectrum should be developed integrating different biometric data (46). Moreover, with the same approach, it would be helpful to identify early disease markers, both for diagnosis and disease relapse. Most AI applications in pediatric oncology have been developed for imaging. However, there is still the need to find novel imaging biomarkers for different types of tumors (43). The efforts to use non-invasive strategies for disease classification are of utmost importance. Indeed, tumors require a histopathologic classification that can be obtained with a surgical approach only. Predicting the prognosis of a tumor from images may help to avoid surgical demolition in low progressing cancers. Through AI it is possible to better classify pediatric tumors and precisely tailor therapeutic approaches to the biology of the tumor and the genotype of the host (47). Personalized therapies and prediction of the impact of genomic variations on the sensitivity of normal and tumor tissue to chemotherapy or radiation therapy are certainly attainable with AI.

AI can also improve the understanding of tumor spread by comparing molecular/anatomical features of primary tumors and metastases or by comparing multiple metastases in the same person and explore genetic, molecular and physiological factors associated with spontaneous tumor regression.

The development of new treatments and drug repurposing is also an important issue (48). Interesting studies with initial evidence in this respect have recently been published (49). More tolerable and safe treatments would significantly improve the quality of life in this population. Among novel treatments, the identification of new opportunities for immunotherapy and the evaluation of combination therapies deserve attention. Moreover, there is a need to rapidly forecast intrinsic resistance and to monitor for the emergence of acquired resistance. Investigating complex pathways to identify combination therapies that minimize the likelihood of disease recurrence, and of genes associated with an increased sensitivity to certain medications or that expose a greater drug toxicity may also help.

Additional opportunities of AI in pediatric oncology are in supporting and accelerating randomized clinical trials for novel therapies, and integrating data from wearable sensors, patient reported outcomes and data from electronic health records.

To achieve these objectives, an acceleration of research efforts is required in the implementation of AI in pediatric oncology at all levels. Most importantly, this effort cannot be pursued in limited geographic areas, but has to be developed at a global scale to take advantage of a collective scientific effort and the largest possible amount of data. The strong international research activities in this field that Europe has been conducting with other continents represent an excellent starting point in this respect.

Additionally, with the exception of some work aimed at applying AI for diagnostic image segmentation, we did not find publications investigating the use of AI to analyze and improve healthcare processes, although we applied a comprehensive search strategy in our review. This observation is in line with the scarce maturity of research in the field of AI in pediatric oncology while future applications may certainly include stratification of patients undergoing invasive procedures and actions that may speed up the patient journey with an impact on quality of care, patient satisfaction, and costs.

Finally, the ethical dimensions of AI in pediatric cancer are of paramount importance not only because of the intrinsic issues of AI, but also because such a technology should be applied in a vulnerable population affected by rare diseases, as cancers in this age group are, severe and potentially fatal, and where genetic information may play an important role. The vast majority of the published literature on AI for pediatric cancer does not address the specificity of these circumstances yet. Notably, a systematic effort on achieving models for a trustworthy AI is ongoing in many international projects (50). It must be underlined that the ethical dimension of AI is multifaceted and includes the explainability of algorithms, their equity, and their safety. These attributes have special implications for children (51) and affect regulatory policies.

## Conclusions

AI has the potential to represent an efficient solution for many unmet needs in pediatric oncology. The work presented in this review shows several potential areas of development and improvement that can match these needs. One central issue for achieving this goal regards the availability of large datasets that should be continuously updated. The future progress of AI in pediatric oncology, as in other fields, will greatly depend on the development of technical solutions that will allow streamline data sharing across different stakeholders while preserving privacy.

## Author Contributions

AT and AM conceptualized the manuscript, coordinated the work, and wrote the manuscript; FF, VD, and EC reviewed the papers included in the analysis, extracted the data, interpreted them, and wrote the results of the review; ME drafted and reviewed the work, and contributed to interpretation of results; IC managed the data analysis and reviewed the results and their interpretation. All authors contributed to the article and approved the submitted version.

## Funding

This paper has been developed with support of the EU4CHILD project that has received funding from the European Union’s Call for Pilot Projects and Preparatory Actions (PPPA) under grant agreement No 101018783.

## Conflict of Interest

The authors declare that the research was conducted in the absence of any commercial or financial relationships that could be construed as a potential conflict of interest.

## Publisher’s Note

All claims expressed in this article are solely those of the authors and do not necessarily represent those of their affiliated organizations, or those of the publisher, the editors and the reviewers. Any product that may be evaluated in this article, or claim that may be made by its manufacturer, is not guaranteed or endorsed by the publisher.
