# Supplementary material for: Gaps and Opportunities of Artificial Intelligence Applications for Pediatric Oncology in European Research: A Systematic Review of Reviews and a Bibliometric Analysis
Source: Front Oncol. 2022 May 31;12:905770. doi: 10.3389/fonc.2022.905770 (PMC9194810; doi:10.3389/fonc.2022.905770)
Supplement: Supplementary file 1 [file DataSheet_1.docx]

***Supplementary Material***

# Supplementary Data

**Appendix 1 - Strategy for literature search**

Search query on Web of Science

(TS=("cancer" OR "oncology" OR "tumor" OR "neoplasm" OR "leukemia" OR "lymphoma" OR "Hodgkin" OR "sarcoma" OR "osteosarcoma" OR "wilms tumor" OR "neuroblastoma" OR "rhabdomyosarcoma" OR "fibrosarcoma" OR "hepatoblastoma" OR "PNET" OR "medulloblastoma" OR "retinoblastoma" OR "glioma" OR "teratom*" OR "myeloproliferative disease" OR "myelodysplastic syndrome" OR "Ependymoma" OR "Carcinoma" OR "Germinom*" OR "Dysgerminom*" OR "bone marrow transplant*" or "stem cell transplant*"))

AND

(TS= (("artificial intelligence" OR "machine learning" OR "deep learning") ) OR TS= (("support vector machine" OR "random forest" OR "Markov decision process" OR "hidden Markov model" OR "fuzzy logic" OR "k-nearest neighbor" OR "naive Bayes" OR "Bayesian learning") ) OR TS=(("artificial neural network" OR "convolutional neural network" OR "recurrent neural network" OR "generative adversarial network" OR "deep belief network" OR "perceptron") ) OR TS= (("natural language processing" OR "natural language understanding") ) OR TS=radiomic*)

Databases= WOS, KJD, MEDLINE, RSCI, SCIELO Timespan=2000-2021

Search language=Auto

Search query on PubMed

(leukemia OR leukemi* OR leukaemi* OR (childhood ALL) OR AML OR lymphoma OR lymphom* OR hodgkin OR hodgkin* OR T-cell OR B-cell OR non-hodgkin OR sarcoma OR sarcom* OR sarcoma, Ewing's OR Ewing* OR osteosarcoma OR osteosarcom* OR wilms tumor OR wilms* OR nephroblastom* OR neuroblastoma OR neuroblastom* OR rhabdomyosarcoma OR rhabdomyosarcom* OR teratoma OR teratom* OR hepatoma OR hepatom* OR hepatoblastoma OR hepatoblastom* OR PNET OR medulloblastoma OR medulloblastom* OR PNET* OR neuroectodermal tumors, primitive OR retinoblastoma OR retinoblastom* OR meningioma OR meningiom* OR glioma OR gliom* OR pediatric oncology OR paediatric oncology OR childhood cancer OR childhood tumor OR childhood tumors OR brain tumor* OR brain tumour* OR brain neoplasms OR central nervous system neoplasm OR central nervous system neoplasms OR central nervous system tumor* OR central nervous system tumour* OR brain cancer* OR brain neoplasm* OR intracranial neoplasm* OR leukemia lymphocytic acute OR leukemia, lymphocytic, acute[mh])

AND

((("artificial intelligence" OR "machine learning" OR "deep learning") ) OR (("support vector machine" OR "random forest" OR "Markov decision process" OR "hidden Markov model" OR "fuzzy logic" OR "k-nearest neighbor" OR "naive Bayes" OR "Bayesian learning") ) OR (("artificial neural network" OR "convolutional neural network" OR "recurrent neural network" OR "generative adversarial network" OR "deep belief network" OR "perceptron") ) OR (("natural language processing" OR "natural language understanding") ) OR radiomics).

**Appendix 2 - Cancer types considered typical of childhood and included in our study**

Acute leukemia; Lymphoma; Hematopoietic stem cell transplantation; Central nervous system tumors; Bone sarcomas; Soft tissue sarcomas; Neuroblastoma; Wilms tumor; Malignant rhabdoid tumors; Germ cell tumors; adrenocortical tumors; Hepatoblastoma; retinoblastoma; Pleuropulmonary Blastoma; pancreatoblastoma
